# Factors affecting the cultural competence of visiting nurses for rural multicultural family support in South Korea

**DOI:** 10.1186/s12912-017-0269-4

**Published:** 2018-01-08

**Authors:** Min Hyun Suk, Won-Oak Oh, YeoJin Im

**Affiliations:** 1 Department of Nursing, CHA University, 30 Beolmal-lo, Bundang-gu, Seongnam-shi, Gyeongghi-do 13496 South Korea; 2 College of Nursing, Korea University, 145 Anam-ro, Seongbuk-gu, Seoul, 02841 South Korea; 3 College of Nursing Science, Kyung Hee University, 26 Kyungheedae-ro, Dongdaemun-gu, Seoul, 02447 South Korea

**Keywords:** Cultural competence, Cultural diversity, Empowerment, Empathy, Community health

## Abstract

**Background:**

With the recent growth of multicultural families in the Korean society, the importance of the role of qualified visiting nurses in the delivery of culturally sensitive health care has grown dramatically. As the primary health care provider for multicultural families enrolled in public community-based health care centers, the cultural competence of visiting nurses is an essential qualification for the provision of quality health care for multicultural families, especially in rural areas. Cultural competence of visiting nurses is based on their cultural awareness and empathetic attitude toward multicultural families. This study aimed to examine the levels of cultural competence, empowerment, and empathy in visiting nurses, and to verify the factors that affect the cultural competence of visiting nurses working with rural multicultural families in South Korea.

**Methods:**

Employing a cross-sectional descriptive study design, data from 143 visiting nurses working in rural areas were obtained. Data collection took place between November 2011 and August 2012. The measurement tools included the modified Korean version of the Cultural Awareness Scale, the Text of Items Measuring Empowerment, and the Interpersonal Reactivity Index to measure the level of empathy of visiting nurses. Analyses included descriptive statistics, a t-test, an ANOVA, a Pearson correlation coefficient analysis, and a multiple linear regression analysis.

**Results:**

The cultural competence score of the visiting nurses was 3.07 on a 5-point Likert scale (SD = 0.30). The multiple regression analysis revealed that the cultural competence of visiting nurses was significantly influenced by experience of cultural education, empathy, and scores on the meaning subscale of the empowerment tool (R^2^ = 10.2%).

**Conclusions:**

Institutional support to enhance visiting nurses’ empowerment by assuring the significance of their job and specific strategies to enhance their empathy would be helpful to improve the cultural competence of visiting nurses. Additionally, regular systematic education on culturally sensitive care would be helpful to enable visiting nurses to provide culturally sensitive care for multicultural families.

## Background

Cultural competence indicates the awareness and incorporation of knowledge about individuals and groups of people into specific standards, policies, practices, and attitudes by incorporating the patient’s views, personal value base and beliefs [1]. Cultural competence in nursing reflects the nurse’s ability to provide individualized culturally sensitive patient care with a respect and an openness to the patient’s social or cultural background [2]. In a multicultural society, the cultural competence of health care professionals can be a reflection of their ability to provide sensitive care for clients with diverse social or cultural backgrounds. This may include an understanding of patients’ cultural heritage, respect for patients’ health beliefs, and an understanding of how other cultural issues may affect the level of care provided [3]. As one of the representative multicultural societies, the equal access to health care and the qualified culturally competent care for those with different cultural and ethnic background have been consistently emphasized in the United States. Culturally competent health care enhances health outcomes through the development of positive relationships between health care providers and clients, and it can reduce ethnic disparities in health care [4, 5].

South Korea has rapidly changed into a multicultural society in the recent decade. The major cause of these rapid changes is international marriage, majority of which have occurred between Korean males residing in rural areas and foreign females immigrants from Vietnam, the Philippines, and China. International marriages accounted for 8.7% of all Korean marriages in 2014 [6]. As most of the multicultural families reside in rural areas, and the productive industries are concentrated in the urban areas in Korea, they have a relatively low socioeconomic status as compared to families comprising members with identical cultural backgrounds. In addition to economic vulnerabilities, new concerns such as lack of access to health care, especially in multicultural families living in rural areas, as well as the high possibility of receiving less optimal qualified health care mainly because of a language barrier in specific health care situations have emerged.

With the social agreement on the need to support multicultural families, especially immigrant women and their children, the “Center of Family Support for Marriage Immigrants” program was initiated in South Korea in 2006, to help them enhance their cultural adaptation by overcoming language barriers and maximizing health care access. This program was then extended to the “multicultural family support system” with an aim to meet the health care needs of families with different cultural backgrounds and to provide them comprehensive health care support by introducing a new health care workforce at the national level.

Visiting nurses, as nursing professionals engaged in working in community public health care centers, provide services for the vulnerable population throughout the life span, including care for elderly persons living alone and young infants and toddlers in low income families. The care of visiting nurses typically includes disease prevention and ensures that community residents engage with direct and indirect health care services, including chronic disease management. Since 2007, they have emerged as professionals who provide health care for multicultural families in the primary health care setting.

In this regard, the visiting nurses’ culturally competent care for multicultural families to overcome their lack of access to health care and to ensure quality health care has become critical. Nurses’ cultural competence influences their ability to provide individualized health care for families with various cultural backgrounds. As a significant attribute of cultural competence, empathy is recognized as one of the factors that strengthens the cultural competencies of health care providers [7, 8] and it is considered a critical predictor of successful cultural integration. Empathy is a basic component of cultural competence, which is expressed through their caring and comprehensive attitude with a consideration on patients’ individual experiences [9, 10]. Empathy in visiting nurses refers to the capability of the health care provider to understand clients’ situations, perspectives, and feelings, and also helps them to communicate and act with multicultural families in a positive manner by actively mediating the attributes of cultural competence [10, 11]. Health care providers’ respectful and careful understanding of clients with different cultural backgrounds is a critical prerequisite in culturally competent care. Therefore, culturally competent care is significantly associated with the empathetic ability of nurses, and nurses’ cultural competence may be revealed through their empathetic communication with the family.

Further, when nurses provide competent care for patients with culturally diverse backgrounds, empowerment is an important attribute in their nursing practice that may influence the provision of culturally competent nursing care [12]. Empowerment may enhance nurses’ cognitive resources and improve their confidence through autonomy [13, 14]. By promoting their own sense of power and heightened motivation, nurses are able to provide competent care and empower others. Thus, empowering the cultural competence of health care providers may also be helpful in the provision of improved health care services for vulnerable populations, such as multicultural families.

In spite of the growing need for health care providers’ cultural competence in community health care, there is a serious lack of thorough investigation on the cultural competence of visiting nurses at the domestic and international level. Recent studies on cultural competence in nursing have focused on assessing cultural competence and examining the predictors of the same at an early stage. Additionally, previous domestic studies on multicultural families have identified the current health care status and the health care needs of multicultural families.

Given that the significance of empowerment and empathy of visiting nurses in building their ability to provide cultural competent care for multicultural families, a study to investigate the factors affecting visiting nurses’ cultural competence by including empowerment and empathy would be meaningful to plan strategies for working with multicultural families. Therefore, the present study aimed to examine the levels of cultural competence, empowerment, and empathy in visiting nurses, who are primary health care professionals for multicultural families in the rural areas of South Korea, and to verify the factors that affect the cultural competence of visiting nurses.

## Methods

### Design and sample

A cross-sectional descriptive study design was used. The target population of this study was Korean visiting nurses. The accessible population was a group of visiting nurses who were enrolled in 23 public health centers situated in Gyeongbuk province, the 2nd largest district except for the metropolitan areas in S. Korea, where 15.5% of multicultural families resided in 2012. Inclusion criteria were being enrolled in a public community health care center and being responsible for the health care services for the residents in the corresponding area. Those in charge of office work in the community health care centers were excluded.

We used G power 3.0 analysis to calculate the most reasonable sample size to secure a reliable interpretation of the study results and to prevent immoderate data collection. Using a constant that was most suitable for a multiple regression analysis (effect size .15, a significance level .05, a power .95), a minimum sample of 107 participants was required. Considering a 20% data wastage rate, we collected data from 156 visiting nurses, and the data from 143 participants was finally used after excluding 13 incomplete or incorrectly responded questionnaires.

### Data collection and ethical considerations

All the data collection procedures in this study conformed to the Declaration of Helsinki. The study posed a low or not more than a minimal risk to the study participants. Data collection was conducted with the approval of the head of the relevant public health care centers, from November 2011 to August 2012. We consider the characteristics of multicultural society in South Korea have not been changed dramatically compared to 5 years ago. This is based on our statistics that the cases of international marriage, which is the main unique characteristic factor to shape the multi-cultural society in our society, has not been increased because of the consolidated restriction on international marriage policy since 2011.

With the cooperation of the team manager for visiting nurses, the research team attended the training center for visiting nurses in the public health care centers. After securing permission of the directors who manage and educate visiting nurses at such centers, we attached a public notice for recruiting participants for this study.

The visiting nurses who expressed willingness to join in this study were enrolled. Informed written consent was obtained from all participants following an explanation of the purpose, methods, risks, and benefits of the study and the rights of participants, such as voluntary participation, and provision of the researchers’ contact information provided. Additionally, the participants were informed that they could withdraw from the study at any time. After completing the self-reported questionnaire, they returned it to the research team member in person. Moreover, the confidentiality of the information obtained was guaranteed by all data collectors and investigators by using code numbers rather than personal identifiers and by keeping the questionnaires locked in the corresponding author’s cabinet.

### Measures

#### Demographic characteristics

To investigate the demographic characteristics of visiting nurses, we used a 7-item questionnaire that included age, marital status, education years, experience as a visiting nurse, and experience of education on culturally competent care.

#### Cultural competence

Cultural competence was assessed using the modified Korean version [15] of the Cultural Awareness Scale (CAS) [16] was used. The CAS was selected as it measures comprehensive perception on the cultural competence of the nurses, such as an awareness of the differences in culture, personal values, beliefs, and biases of the self and others, rather than asking about specific knowledge or ability related to the specific cultural areas. Additionally, the items of the measurement tool were adequate for the Korean situation of the newly introduced multicultural society, owing to which nurses’ cultural competence would be at an early stage of development.

After excluding the items related social welfare area and those which were not fit for the Korean situation, a total 12 items were answered on a 5-point Likert scale. It includes three dimensions: “Awareness of the differences in culture,” “Awareness of personal values, beliefs and biases of the self,” and “Awareness of the values, beliefs and biases of others.” Higher scores reflect higher cultural competence. The internal consistency coefficient, as measured by the Cronbach’s alpha, was .73 in the study by Min & Lee [15], and .74 in the present study.

#### Empowerment

The level of empowerment of visiting nurses was measured using the revised Korean version [17] of the Text of Items Measuring Empowerment (TIME), developed by Spreitzer [18]. This measurement focuses on the nurses’ psychosocial empowerment rather than their practice outcomes. We selected this measure because the level of psychosocial empowerment of nurses can enhance their motivation to provide culturally competent care in their daily practice. Additionally, the translated version was suitable enough to use with a Korean sample. A 5-point Likert scale is used to assess each of the 12 items that cover four subscales (“meaning,” “competence,” “self-determination,” and “impact”), with three questions in each subscale. Higher scores reflect higher levels of empowerment. The Cronbach’s alpha of the Korean version was .899 in Nam and Park’s study [17] and .870, 850, and .890 for the subscales in the present study.

#### Empathy

The Korean translation [19] of the Interpersonal Reactivity Index (IRI) used in Davis’ study [20] was used to assess the level of empathy among visiting nurses. It was originally developed to measure empathetic abilities and interpersonal interactions of nursing students. We selected the IRI as it could reflect the visiting nurses’ empathy and sensitive interpersonal relationships in caring for patients with different cultural backgrounds in the Korean society. The index uses a 5-point Likert scales ranging from “Don’t agree” (1) to “Strongly agree” (5) for each of the 28 items. These items cover the following four subscales: “Perspective taking,” “fantasy,” “empathic concern,” and “personal distress.” Content and face validity were assessed by seven health care professionals, including professors in nursing science, doctoral students in nursing, nurse managers, and staff nurses. Two items that did not accurately convey the intended meaning were corrected as a result of this process. The Cronbach’s alpha in a previous study [19] was .84 and .71 in the present study.

### Data analysis strategy

Data analysis was performed using PASW software (version 19.0). Percentages, means, and standard deviations (SDs) were calculated for participants’ demographic characteristics. In addition, means, SDs, and possible ranges of cultural competence, empowerment, and empathy scores were also calculated. Differences in cultural competence scores by demographics were analyzed by either t-tests or ANOVA. Pearson correlation coefficients were computed to examine the relationships among these variables. Finally, each variable that was significantly correlated with scores in cultural competence and the two main research variables (empathy, empowerment) were included in a stepwise multiple linear regression model to verify the factors affecting the cultural competence of visiting nurses.

## Results

Data from the 143 participants were finally analyzed after excluding 13 incomplete questionnaires from the sample of 156 visiting nurses.

### Demographic characteristics and differences in cultural competence by demographics

The demographic characteristics of the sample have been reported in Table 1. The mean age of visiting nurses was 39.44 years (SD = 7.66) and most participants (*n* = 114, 80.3%) were married. The average years of experience as a visiting nurse was 30.12 months (SD = 25.62). Most (79%) had completed a 3-year nursing college qualification and 18.2% had obtained a bachelor’s degree from a nursing school. In total, 90.9% of visiting nurses reported an experience of caring for multicultural families. Overall, 20.3% indicated that they had received training or education on diverse cultural issues that may be helpful when caring for multicultural families.

**Table 1:** Demographic Characteristics of Subjects (*N* = 143)

Characteristics	Categories	n (%)	Mean ± SD
Age (years)	< 30	32 (22.4)	
	30–40	43 (30.1)	39.44 ± 7.66
	> 40	68 (47.6)	
Marital status	Unmarried	29 (19.7)	
	Married	114 (80.3)	
Total experience as a nurse (years)	≤ 3	18 (12.6)	
	3–10	76 (53.2)	9.73 ± 5.84
	> 10	49 (34.2)	
Experience as a visiting nurse (months)	≤ 12	24 (16.8)	
	13–24	35 (24.5)	30.12 ± 25.62
	≥ 25	84 (58.7)	
Education	College	113 (79.0)	
	University	26 (18.2)	
	Master	4 (2.8)	
Experience of caring multicultural family	Yes	130 (90.9)	
	No	13 (9.1)	
Experience of cultural education	Yes	29 (20.3)	
	No	114 (79.7)	

Significant differences in visiting nurses’ cultural competence scores were revealed between those that had undergone education on multicultural nursing and those that had not (*t* = 2.53, *p* = .012), with the former exhibiting higher cultural competence scores (Table 2).

**Table 2:** Differences in Cultural Competency by Demographic Characteristics (*N* = 143)

Characteristics	Categories	Cultural competence		
		Mean (SD)	*t/F*	*ρ*
Age (years)	< 30	36.56 (3.33)	0.48	0.621
	30–40	36.74 (4.09)		
	> 40	37.15 (3.36)		
Marital status	unmarried	36.44 (4.09)	1.10	0.271
	married	37.17 (3.53)		
Total experience as a nurse (years)	≤ 3	37.77 (3.31)	2.04	0.132
	3–10	36.22 (3.82)		
	> 10	36.57 (3.25)		
Experience as a visiting nurse (months)	≤ 12	37.44 (2.93)		
	13–24	35.96 (3.12)	2.48	0.087
	≥ 25	37.10 (3.94)		
Education	College	36.89 (3.61)		
	University	37.14 (3.75)	1.06	0.348
	Master	35.07 (3.14)		
Experience of caring for multicultural families	Yes	36.82 (3.66)		
	No	37.20 (2.64)	0.36	0.722
Experience of cultural education	Yes	38.12 (3.20)	2.53	0.012^*^
	No	36.54 (3.64)		

*Significant at *p* < .05

### Cultural competence, empowerment, and empathy in visiting nurses

The average cultural competence score among visiting nurses was 36.84 (SD = 3.61), with an item mean of 3.07 (SD = 0.30) (Table 3). The subscales showing the highest scores were awareness of personal values, beliefs, and biases of the self (Mean = 3.15, SD = 0.42) and awareness of the differences in culture (Mean = 2.98, SD = 0.45), respectively.

**Table 3:** Descriptive Statistics for the Study Variables (*N* = 143)

	Mean	SD	Item mean	Item SD	Range
Cultural competence (total)	36.84	3.61	3.07	0.30	60–31
Differences in culture	11.93	1.82	2.98	0.45	20–8
Personal values, beliefs and biases of selves	9.46	1.27	3.15	0.42	15–6
Values, beliefs, and biases of others	15.45	1.91	3.09	0.38	25–13
Empowerment (total)	44.95	6.47	3.75	0.54	59–29
Meaning	12.71	1.98	4.24	0.66	15–8
Competence	12.03	1.90	4.01	0.63	15–8
Self-Determination	11.29	2.39	3.76	0.80	15–5
Impact	8.92	2.65	2.97	0.88	15–3
Empathy	150.21	10.15	3.58	0.36	163–81

The average empowerment score of visiting nurses was 44.95 (SD = 6.47), with an item mean of 3.75 (SD = 0.54). The subscale with the highest item mean was meaning (Mean = 4.24, SD = 0.66), followed by competence (Mean = 4.01, SD = 0.63), self-determination (Mean = 3.76, SD = 0.80), and impact (Mean = 2.97, SD = 0.88), respectively. The average empathy score of the participants was 150.21 (SD = 10.15), with an item mean of 3.58 (SD = 0.36) on a 5-point Likert scale.

### Correlations among primary variables

Correlations among cultural competence, empowerment, and empathy have been presented in Table 4. The total mean score of cultural competence in visiting nurses were positively correlated with the meaning (*r* = .17, *p* = .016) and competence (*r* = .16, *p* = .022) subscales of empowerment. The higher the mean scores on the meaning subscales of empowerment were, the higher were the mean scores on the “awareness of differences in culture” subscale of cultural competence (*r* = .16, *p* = .018). Further, positive correlations were found between the cultural competence subscale of awareness of personal values, beliefs, and biases of the self and the empowerment subscales of meaning (*r* = .16, *p* = .025) and competence (*r* = .14, *p* = .042). Although there was no significant correlation between the overall cultural competence score and empathy, there was a significant positive correlation between the cultural competence subscale of awareness of personal values, beliefs, and biases of the self and overall empathy (*r* = .15, *p* = .035).

**Table 4:** Correlation Coefficients of the Study Variables (*N* = 143)

	CC	CC-D	CC-P	CC-O	Emp	Emp-Mean	Emp-Comp	Emp-Self	Emp-Imp	Empathy
Cultural Competence (CC)	1									
Differences in culture (CC-D)	0.71 (<.000)^**^	1								
Personal values, beliefs and biases (CC-P)	0.68 (<.000)^**^	0.28 (<.000)^**^	1							
Values, beliefs, and biases of others (CC-O)	0.76 (<.000)^**^	0.20 (0.003)^**^	0.35 (<.000)^**^	1						
Empowerment (Emp)	0.12 (0.081)	0.09 (0.203)	0.11 (0.129)	0.07 (0.315)	1					
Meaning (Emp-Mean)	0.17 (0.016)^*^	0.16 (0.018)^*^	0.16 (0.025)^*^	0.06 (0.421)	0.65 (<.000)^**^	1				
Competence (Emp-Comp)	0.16 (0.022)^*^	0.08 (0.269)	0.14 (0.042)^*^	0.13 (0.055)	0.74 (<.000)^**^	0.54 (<.000)^**^	1			
Self-determination (Emp-Self)	0.08 (0.231)	0.05 (0.502)	0.09 (0.193)	0.05 (0.452)	0.81 (<.000)^**^	0.38 (<.000)^**^	0.48 (<.000)^**^	1		
Impact (Emp-Imp)	0.02 (0.783)	0.00 (0.959)	0.03 (0.688)	0.21 (0.837)	0.69 (<.000)^**^	0.11 (0.107)	0.25 (0.000)^**^	0.45 (<.000)^**^	1	
Empathy	0.03 (0.663)	0.02 (0.770)	0.15 (0.035)*	0.06 (0.396)	0.21 (0.003)*	0.19 (0.006)*	0.26 (0.002)^**^	0.11 (0.120)	0.14 (0.051)	1

*Significant at *p* < .05

**Significant at *p* < .01

Finally, empathy was found to have a significant positive correlation with empowerment (*r* = .21, *p* = .003), meaning (*r* = .19, *p* = .006), and competence (*r* = .26, *p* = .002).

### Factors affecting the cultural competence of visiting nurses

The variables included in the multiple regression analysis used to identify the factors affecting the cultural competence of visiting nurses were empowerment, empathy, and the experience of education on cultural issues, which was the demographic variable showing a statistically significant difference in cultural competence scores. As cultural education experience was measured through a yes/no question, this was entered as a dummy variable in the multiple regression analysis.

Multicollinearity and residual analyses were conducted prior to the multiple regression analysis. All tolerance parameter estimates were over 0.1, and they ranged from 0.63–0.96. The values of the variance inflation factors (VIFs) were 1.04–1.70, which may have ruled out any issues associated with problems of multicollinearity among the variables included in the multiple regression. A subsequent residual analysis revealed a Durbin-Watson value of 1.89, which verified the independence among residuals. All 143 observed values were included, as the Cook’s D values were less than 0.1 for all participants.

In the subsequent multiple regression analysis that was conducted to identify the relative influences of the study variables, experience of cultural education, empathy, and the meaning subscale of empowerment were found to have a significant influence on the cultural competence of visiting nurses. The R square of the regression model was 10.2%, and it was 4.3% for cultural education experience, 3.85% for empathy, and 2.1% for meaning. These figures have been presented in Table 5.

**Table 5:** Factors Affecting Cultural Competence (*N* = 143)

Variables	B	S.E.	*β*	Adjusted *R*^2^	Cumulative *R*^2^	*t (ρ)*	*F (ρ)*
Experience of cultural education	2.67	0.82	0.17	0.043	0.043	6.99 (.009)^**^	7.61 (<.001)^**^
Empathy	0.04	0.02	0.02	0.038	0.081	6.45 (.012)^*^	
Empowerment (meaning)	2.17	0.17	0.17	0.021	0.102	4.75 (.031)^*^	

*Significant at *p* < .05

**Significant at *p* < .01

## Discussion

This study investigated the cultural competence of visiting nurses in Korea and identified the factors that affect this trait by including the variables of empowerment and empathy.

First, we could verify that the visiting nurses were primarily responsible for providing care to multicultural families in the rural area in which the present study was conducted. Specifically, over 90% of the visiting nurses in this study had experienced the provision of care for multicultural families. In spite of the rapid growth of multicultural families in the recent decade, there has been rudimentary level of governmental support for health care professionals in providing culturally sensitive care for multicultural families, which was evidenced by the finding that only about 20% of the visiting nurses had received training on culturally sensitive care.

In other countries with a large multicultural population, such as the United States, cultural diversity training is integrated with the nursing curriculum and forms a part of their clinical nursing practice [21, 22]. Such a system helps to ensure that health care professionals have adequate cultural competence and cultural sensitivity to successfully work with multicultural families and communities [23]. In Korea, a developing multicultural society, there has been a lack of preparation for the various challenges associated with multiculturalism and this is also true for the application of health care and the nursing curriculum in general.

Therefore, it will be important that such gaps in the system be addressed and that cultural diversity issues are discussed and acted upon. A systematic review reported that educational programs to improve nurses’ culturally competent care have a positive influence on nurses’ cultural competence [24]. Therefore, a well-designed education program aimed to enhance the cultural competence of visiting nurses, and which is suitable enough to reflect the Korean cultural context, should be introduced as early as possible.

Although a direct comparison of study results may be limited due to the shortage of studies on multicultural issues, the mean cultural competence scores among visiting nurses in this study were above the median level, which was higher than those among nurses in tertiary level university hospitals [25] and undergraduate nursing students in Korea [26] but were lower than those in undergraduate freshmen and faculty members in US nursing schools [27]. Considering that nurses’ cultural competence is reflected in their ability to provide qualified health care to clients with various cultural backgrounds [12], it was significant to find that levels of empowerment among visiting nurses in this study were above the median value. However, as compared to scores on the meaning and competence subscales of empowerment, those on the impact subscale of this measure were relatively low. This means that although nurses may easily perceive the importance of providing health care to families and are relatively confident in doing so, they tend to downplay the importance of their specific role in health care outcomes, possibly due to their unstable position as an employee. Additional institutional support aimed at providing increased job security for visiting nurses might help enhance their empowerment.

It is significant to note the present findings of empathy scores above the median level. Empathy allows visiting nurses to create interactions based on mutual respect and through the acceptance of alternative lifestyles and the reduction of bias [28, 29]. Nurses with high level of cultural competence are able to use interpersonal communication, relationship skills, and behavioral flexibility to work effectively in cross-cultural situations [30].

In regard to correlation among study variables, there was no significant correlation between empowerment and cultural competence, which resembles the results reported by Bauce, Kridli, and Fitzpatrick [12]. Given that empowerment may be an important contributor to professional nursing practice and may influence the provision of culturally competent care [12], strategies to enhance nurses’ empowerment, which subsequently may strengthen culturally sensitive care, need to be developed. Instrument development to measure nurses’ empowerment in regard to their culturally competent care would also be helpful for further investigation.

Further, contrary to previous studies, we could not find a significant correlation between empathy and cultural competence in this study. This may have occurred owing to the use of the IRI to assess empathy, as it focuses on the broad aspects of nursing, and not the aspects of perception, attitude, and specific skills of culturally competent care in nurses. Development of a sensitive instrument to measure nurses’ empathy in the Korean culture is therefore necessary. Nonetheless, empathy was finally verified as one of the factors influencing the cultural competence of visiting nurses in our regression model.

As an important finding, we identified the three significant factors affecting the cultural competence of visiting nurses in this study, namely, cultural education experience, empathy, and perceived work role meaning (a subcategory of empowerment). First, improved cultural competence among health care providers has a positive effect on patient care by creating interactions that are based on respect and that incorporate the health beliefs, practices, and cultural and linguistic needs of culturally diverse clients. Considering the current lack of training on cultural issues available to visiting nurses, the introduction of culture-related education is an urgent issue in Korea’s new multicultural society. For multicultural families in rural areas, considered as one of the vulnerable sections of the populations in terms of health care access and quality, careful investigations into their current health care status and future health needs are also required in order to provide effective health care. Additionally, the development and implementation of strategies to enhance empathetic attitudes and levels of empowerment among visiting nurses, especially those related to the importance of their own roles as health care providers, are required to improve the cultural competence of visiting nurses.

In spite of the significant research findings of this study that identified the factors that affect cultural competence among visiting nurses, there were some limitations in this study.

First, the population in this study included a limited location of Korean rural areas in 2011/2012. Although we calculated the sample size based on G power analysis to secure reliable interpretation of the study results, and the study location was wide enough to ensure the selection of study participants with a suitable effect size, a nationwide study would be beneficial to confirm the present results. Given the dynamic nature of migration and research in this area, it is important to acknowledge the findings relate one point in time.

Second, the regression model in this study reported a relatively low R square value. This indicates that an additional study would require to incorporate other variables that may also influence cultural competence, such as individual knowledge on cultural issues, linguistic competence, ideas on patient centeredness, and/or degree of understanding of the health care system [31, 32]. Additionally, it would be beneficial to use other cultural competence measurements after assessing the psychometric properties of the Korean translated version of the tools.

## Conclusions

Cultural education experience, empathy, and perceived work role meaning as a subcategory of empowerment were found to have a significant influence on the cultural competence of visiting nurses. Considering the rapid social changes leading to a culturally-diverse health care environment, it is critical to develop appropriate educational programs for visiting nurses, who are the foremost nursing profession caring for culturally diverse patients in the rural community. In this context, practical and efficient educational protocols should include methods to foster the self-awareness of visiting nurses on their potential biases toward the patient with different cultural backgrounds. Additionally, attention should be paid on the improvement of the nurses’ empathetic and accepting attitude by enhancing their understanding of different health care customs. Second, efforts to enhance the mutual communication between visiting nurses and patients are required. Active involvement of the health care system to decrease language barriers, such as commercialization of phone or in-person interpreters, needs to be designed. Finally, we need to reinforce the general empowerment of visiting nurses, which would subsequently enable them to provide competent nursing care for culturally diverse patients.

## Abbreviations

CC: Cultural competence
CC-D: Cultural competence-differences in culture
CC-O: Cultural competence-values, beliefs, and biases of others
CC-P: Cultural competence-personal values, beliefs and biases
Emp: Empowerment
Emp-Comp: Empowerment-competence
Emp-Imp: Empowerment-impact
Emp-Mean: Empowerment-meaning
Emp-Self: Empowerment-self-determination
SD: Standard deviation
